# A Radiographic and Clinical Comparison of Immediate vs. Early Loading (4 Weeks) of Implants with a New Thermo-Chemically Treated Surface: A Randomized Clinical Trial

**DOI:** 10.3390/ijerph18031223

**Published:** 2021-01-29

**Authors:** Matteo Albertini, Federico Herrero-Climent, Carmen María Díaz-Castro, Jose Nart, Ana Fernández-Palacín, José Vicente Ríos-Santos, Mariano Herrero-Climent

**Affiliations:** 1 Department of Periodontology, Universitat International de Catalunya, 08017 Barcelona, Spain; malbertini@uic.es (M.A.); josenart@uic.es (J.N.); 2 Clinica Dental Herrero Climent, Calle del Dr. Fleming 54, 28036 Madrid, Spain; clinica@fherrerocliment.net; 3 Department of Preventive Medicine and Public Health, Faculty of Medicine, Universidad de Sevilla, Avda Sanchez Pizjuán S/N, 41009 Sevilla, Spain; carmmaria@hotmail.com (C.M.D.-C.); afp@us.es (A.F.-P.); 4 Department of Periodontics and Dental Implants, School of Dentistry, University of Seville, C/Avicena S/N, 41009 Sevilla, Spain; 5 Porto Dental Institute, 4150-518 Porto, Portugal; dr.herrero@herrerocliment.com

**Keywords:** immediate loading, early loading, dental implants, bone–implant interface, osseointegration

## Abstract

**Background:** Implant dentistry has evolved over time, resulting in better treatment outcomes for both patients and clinicians. The aim of this trial was to test whether the immediate loading of implants with a platform-switching design influences the marginal bone level, compared to four-week loading, after one year of follow-up. Moreover, a comparison of clinical data regarding implant survival, implant stability, and patient-reported outcome measures (PROMs) was conducted. **Methods:** Klockner^®^ VEGA^®^ implants with a ContacTi^®^ surface were placed in partially edentulous patients in the posterior areas. Group A received an immediately loaded prosthesis (one week) and Group B received an early-loaded prosthesis (four weeks). All abutments were placed at the time of surgery. Radiographic and clinical data were recorded. **Results:** Twenty-one patients were treated (35 implants). No implants were lost during the study. The final marginal bone level did not show differences between groups. The bone loss at 12 months at the implant level was 0.00 mm for both groups (median). The final implant quotient stability (ISQ) values did not differ between groups (median 73 and 70.25), nor did the other clinical parameters or PROMs. **Conclusions**: The results suggest that neither of the loading protocols with the implants used influenced the marginal bone level—not the osseointegration rate, clinical conditions, or PROMs.

## 1. Introduction

Implant dentistry has evolved in recent decades, resulting in better treatment outcomes for both patients and clinicians. This improvement has been possible through progressive changes in each and every aspect of therapy: implant design, implant surface, prosthetic connections, abutments, materials, surgical and loading protocols, and so on [1]. In this sense, shortening the period to treatment completion has been always a main concern, including consideration of the immediate installation of the prosthesis, and, consequently, the immediate loading of the implants [2,3]. Immediate loading is defined as the connection of the restoration onto the implant in occlusion with the opposing dentition within the first week after implant placement; in contrast, early loading is considered when prosthesis installation takes place between the first week and two months; finally, conventional loading refers to installation that takes place later on [4,5]. Predictable results have been reported in terms of implant survival rates and marginal bone loss, with both immediate and early loading procedures, under some specific clinical conditions, mainly in the anterior region [4,6]. Even in the posterior areas, several clinical studies have shown a high implant success rate in the presence of an adequate primary stability and favorable occlusal conditions under an early loading approach [7,8] and, moreover, under immediate loading [9].

Considering the factors involved in implant osseointegration, it is generally accepted that the implant surface plays a major role regarding the bone healing process around dental implants. In fact, from the early days of osseointegration, the development of new surfaces has been a main issue in terms of promoting a greater rate of osseointegration, a faster bone apposition, and greater bone–implant contact [1,10]. In the field of implant surfaces, one of the proposals to reach these goals has been the modification of the endosseous implant surface by the deposit of a layer of diverse so-called bioactive molecules, such as hydroxyapatite (HA), bioactive glasses, and calcium phosphate [11,12,13]. Different manufacturing processes, including the plasma spray technique, have enabled manufacturers to obtain implants with an HA-coated surface onto titanium or titanium alloy implant bodies [14,15]. Although these implants, years ago, showed promising results in the short term regarding bone healing [16,17], diverse complications were later reported, mainly due to failures of adhesion of the HA coating, leading, finally, to bacterial microleakage and peri-implant tissue complications [13,18,19]. In 1990, Kokubo et al. proposed a titanium surface modification by means of an initial chemical treatment with alkaline solutions and a subsequent heating process at high temperatures [20]. This method has demonstrated de novo HA formation through a biomimetic reaction once the implant is in contact with the human serum, without the presence of osteoblasts [21,22]. In vitro experiments in a simulated body fluid model have demonstrated, by electromicroscopy, new deposits of apatite particles in implants with this surface modification [21,23,24,25]. This method has also shown de novo HA formation once the implant is in contact with human serum, without the presence of osteoblasts [21,22]. Authors have suggested that the crystalline apatite layer adhered to titanium has the potential to stimulate osteoblast migration in contact with the living bone, and, consequently, to accelerate the osseointegration process [23].

Furthermore, in vivo studies have observed de novo bone formation and increased bone–implant contact in the early stages of bone healing, in comparison to non-treated surfaces. Based on these investigations, a highly hydrophilic and osteoconductive surface was developed [26,27] and has been applied to dental implants for its clinical use [28], under the commercial name of ContacTi^®^ (Klockner Implant System, SOADCO S.L., Andorra). As already described by Aparicio et al. [29], once the implants are produced in commercially pure titanium grade 4, their surface undergoes a process of grit-blasting and acid-etching in order to obtain a moderately rough surface, followed by thermo-chemical modification. A recent clinical investigation with a bioactive HA surface showed promising results with both immediate and early loading [30].

Regarding implant morphology, years ago, the platform-switching design was introduced, meaning that the diameter of the abutment placed onto an implant is narrower than the inner wall of the implant itself [31]. The use of platform-switching has been claimed to protect from bone loss around implants and, as a consequence, to facilitate the maintenance of peri-implant tissue health [32,33]. In addition, whether repeated implant abutment disconnections may lead to marginal bone resorption has also been investigated, but this is, at least at the present moment, still a matter of controversy [34,35,36]. Recent studies have suggested that one single abutment connection at the time of implant surgery, the so-called one abutment–one time (OAOT), would favor peri-implant marginal bone level maintenance [37,38,39]. Under the platform-switching design, a new implant model has been marketed, named Klockner^®^ VEGA^®^ ContacTi^®^ (Vega-CTi) (SOADCO S.L., Andorra), featuring: (a) a 10° Morse taper internal conical connection, (b) an internal hexagon for index and anti-rotation purposes, (c) a platform-switching design, and (d) enhanced titanium, i.e., grade 4 titanium cold worked at high tension. The implant surface is the already mentioned and previously described ContacTi^®^.

Considering the previous statements, a clinical protocol was developed to investigate the clinical performance of Vega-CTi implants. Therefore, the aim of this randomized clinical trial was, primarily, to test the null hypothesis that the immediate loading of Vega-CTi implants (less than one week) would not influence the marginal bone level, compared to four-week loading, under the concept of OAOT, one year after treatment completion. Additionally, this study obtained and compared clinical data regarding implant survival, implant stability values, and the patient-reported outcome measures (PROMs) under these loading conditions.

## 2. Materials and Methods

The present study was originally designed as a prospective, multi-center, single-blinded, controlled clinical trial. This article presents the one-year results of the trial.

### 2.1. Participants

Adult patients, at least 18 years old, missing at least one posterior tooth (molar or premolar) in the mandible or maxilla and willing to receive implant therapy were eligible for screening. In addition, the following inclusion criteria were required:

- Undisturbed, completely healed edentulous sites, at least four months after tooth/teeth extraction;
- Adequate bone volume verified by CBCT (cone beam computed tomography) for the placement of a dental implant of at least 8 mm in length and 3.5 mm in diameter; this means at least 9 mm of bone in the vertical dimension and 6.5 mm in the horizontal dimension;
- No bone regeneration procedures required;
- Adequate prosthetic conditions for proper crown or bridge installation;
- Natural antagonist dentition, whether restored or untreated teeth;
- Presence of at least 4 mm of keratinized mucosa from the buccal to lingual side of the edentulous ridge;
- Absence of oral infections, gingival inflammation, untreated periodontitis, or mucosal diseases.

### 2.2. General Exclusion Criteria

The general exclusion criteria included:

- Uncontrolled systemic diseases;
- Pregnancy;
- Bone-related disorders such as osteoporosis;
- Drug abuse or alcoholism;
- Smoking habit of >10 cigarettes per day;
- Severe bruxism or severe temporomandibular junction disorders;
- Previous history of implant failure.

In order to enter the study, patients had to be able to understand the nature of the study, to sign an informed written consent form, and to be willing to attend the scheduled follow-up appointments. This study was conducted in accordance with the Declaration of Helsinki (1964) and its amendments for patients participating in clinical studies (Tokyo, 1975; Venice, 1983; Hong Kong, 1989; Somerset, 1996; Edinburgh, 2000). The study protocol was reviewed and approved by the ethics committee for clinical studies of the Universitat Internacional de Catalunya with reference number 500077.

### 2.3. Location and Clinicians

Three centers in Spain (Barcelona Dental Institute Barcelona, the CEROM Clinic in Marbella, and the NCD Clinic in Barcelona) participated in the study. Three experienced dentists, one at each center, all of them familiarized with the implant system, were responsible for the surgical and prosthetic procedures (M.A., M.H., and J.N.). Before the study started, the clinicians participated in a calibration meeting to receive all the information (verbally and in writing) about the study and the assessment of the variables.

### 2.4. Implants and Abutments

Vega-CTi implants, as already described, of 3.5, 4.0, and 4.5 mm in diameter and 8, 10, and 12 mm in length were available for the surgeries. The dimensions of the platform-switching for each implant diameter were 0.3, 0.35, and 0.6 mm, respectively. All of the restorations were installed onto abutments of 1 or 2 mm in transmucosal height, designed for screw-retained prostheses (Permanent^®^ Abutment, Klockner Implant System, SOADCO S.L., Andorra). No cemented restorations were used in this study.

### 2.5. Pre-Treatment Procedures

All clinicians were asked to follow a common pre-treatment protocol, including intra-oral photographs, impressions, cast models, diagnostic wax-up, intra-oral radiographs, orthopantomography, and cone beam computerized tomography. With all of this information, prosthetically guided implant placement was planned.

The patients received teeth prophylaxis, including oral hygiene instructions, full-mouth plaque scores (FMPS), and full-mouth bleeding scores (FMBS). The periodontal chart was completed recording the clinical attachment level (CAL) and probing depth (PD) with a conventional periodontal probe (PCP UNC 15-Hu-Friedy). Periodontal status was assessed according to the classification proposed by Armitage 1999 [40].

### 2.6. Surgical Interventions

The patients received prophylactic antibiotic therapy 1 h prior to surgery (1 g of amoxicillin or 600 mg of clindamycin in case of allergy or intolerance). The patients were told to mouthwash for 1 min with a 0.12% solution of chlorhexidine (Perio Aid^®^, Dentaid, Spain); later, a 2.0% solution of chlorhexidine (Bohmclorh^®^, Bohm, Madrid, Spain) was applied extra-orally to the peri-oral skin. The surgeries were performed under local anesthesia with 40 mg/mL of articaine + 0.01 mg/mL of epinephrine (Artinibsa^®^, Inibsa Dental, Barcelona, Spain). Then, the full-thickness flaps were raised by means of crestal and intrasulcular incisions, paying attention to preserving at least 2 mm of keratinized mucosa on the buccal and lingual or palatal aspects of the alveolar crest. The crestal bone width was measured buccolingually with a periodontal probe (PCP-UNC 15, Hu-Friedy). The bone site preparation was performed according to the manufacturer’s drilling sequence suggested for Vega-CTi implants; however, each clinician was free to adapt the final drilling according to the bone quality in order to ensure optimal primary stability. The implants were inserted using a calibrated torque wrench (Klockner Implant System, SOADCO S.L., Andorra), taking care to place the implant shoulder at least 1 mm below the crestal level at the most declining part of the bone preparation. Each clinician could decide the optimal diameter and length of the implants, considering the preoperative diagnostic procedures and the surgical evaluation of the bone volume available at the site. Bone quality was registered according to the D1-, D2-, D3-, and D4-type classifications [41]. Insertion torque (IT) and primary stability were registered after implant placement as follows:

(A) Manually, by means of a calibrated torsion torque wrench (Klockner Implant System, SOADCO S.L., Andorra) at the time of placing the implant.
(B) Manually, after the use of the torque wrench, according to the following criteria at the time of the abutment connection: Type A: IT > 35 N/cm, and the implant could not be rotated manually; type B: IT < 35 N/cm and the implant could not be rotated manually; type C: IT < 35 N/cm and the implant could be rotated manually; type D: the implant could be rotated manually and moved in a vertical direction. In this case (i.e., type D), the patient was excluded from the study, and a delayed loading protocol was carried out.
(C) By radiofrequency, using the Osstell^®^ device and Osstell^®^ transducers (Smart-Pegs^®^) (Osstell^®^, Gothenburg, Sweden) in order to obtain of the implant stability quotient (ISQ) values. The transducers were connected to the abutments, screwed at 5 N/cm.

After implant installation, the straight abutments for screw-retained restorations were tightened at 20 N/cm with a dynamometric wrench (Klockner Implant System, SOADCO S.L., Andorra). The selection of the height of the abutment (1 or 2 mm) was subject to the criteria of the clinician at the time of surgery. The ISQ values for the implants in the study were recorded, both buccolingually and mesiodistally. Abutment-level impressions were taken the same day of the surgery, and screw-retained acrylic provisional restorations were prepared for all implants. Finally, healing caps were placed onto the abutments and the surgical flaps were sutured with 5/0 polyamide avoiding any tension. Antibiotic treatment was maintained for seven days (500 mg of amoxicillin or 300 mg of clindamycin for 1/8 h). Non-steroidal anti-inflammatory dexketoprofen (25 mg) was administrated every 8 h if needed, as was a gastric protector (20 mg of pantoprazole) once per day for seven days. At this point, a randomization process was performed in order to allocate the patients to Group A, in which the implants were loaded with provisional restorations within the first week after surgery, or Group B, in which the implants were loaded four weeks after surgery. The randomization was done by the toss of a coin, by an assistant who was not involved in the investigation.

### 2.7. Prosthetic Procedure

Provisional prostheses up to three units and with no cantilever or implant–tooth junctions were inserted and tightened at 20 N/cm within the first week after implant placement in patients of Group A and after four weeks in patients of Group B. Eight weeks after implant placement, the provisional restorations were unscrewed, and definitive impressions were taken. All definitive prostheses (also up to three units and with no cantilever or implant–tooth junctions) were delivered 10–12 weeks later; the abutments were re-tightened at 30 N/cm and restoration screws at 25 N/cm with a dynamometric wrench. The occlusal screw access hole was filled with a composite material and occlusal adjustment and polishing were carried out if needed. All of the restorations, whether provisional or final, were in occlusion with the opposite dentition in the maximum intercuspation position. If there were any complications with the prostheses, they were recorded (screw loosening, abutment loosening, fracture, chipping, etc.).

### 2.8. Outcomes

The primary outcome measure was the peri-implant marginal bone level changes. For this purpose, standardized periapical radiographs were taken at five time points: immediately after implant placement (baseline), after the temporary restoration installation (at one or four weeks), after definitive restoration installation (at 10–12 weeks), and at 6 and 12 months. Baseline radiographs were taken with the definitive abutment and the corresponding healing cap screwed. Radiographs for assessing the impression coping fit and the prosthetic components were also performed, but only for clinical purposes. Once the final crowns were in place, a customized standard holder was prepared for each implant, recording the occlusal surfaces of the adjacent and opposing teeth with acrylic resin on a parallel X-ray holder (Dentsply Rinn XCP-DS^®^) in order to obtain a consistent image projection. An independent blinded technician performed the radiographic analysis. A previous calibration procedure was carried out as follows: The same examiner measured a subset of 10 radiographs on three separate occasions, 72 h apart, to determine the intra-examiner reproducibility. An interclass coefficient of 0.99 (*p* < 0.05) was obtained. The radiographic peri-implant bone level changes in the X-rays were analyzed using software for image analysis (ImageJ^®^, version 1.39F, U.S. National Institutes of Health). All measurements were performed using 7X magnification.

**Figure 1:**
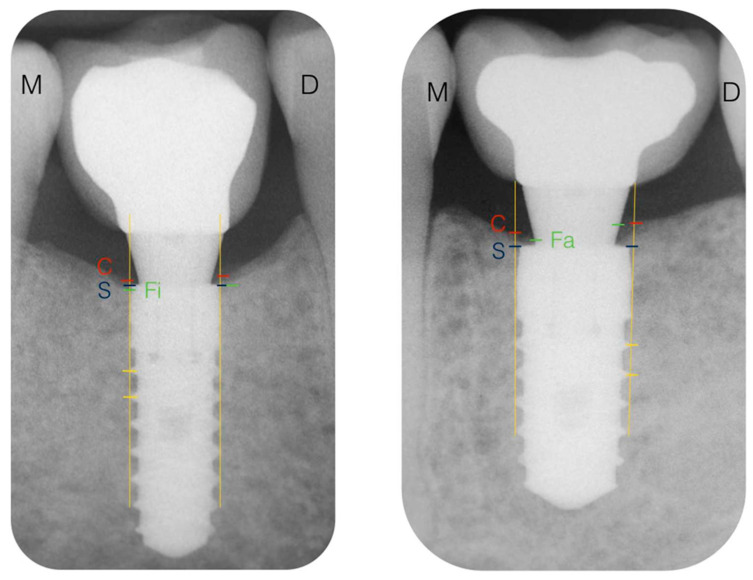
Reference landmarks in the X-rays. C, bone crest; S, implant shoulder; Fi, first bone–implant contact; M, mesial; D, distal; S–C distance, marginal bone level at the crest (MBLc); S–Fi distance, marginal bone level at the implant (MBLi); S–Fa distance, marginal bone level at the abutment (MBLa).

Figure 1 shows the reference landmarks for the X-ray analysis. In each radiograph, the most coronal part of the bone crest (C), the position of the implant shoulder (S), and the level of the first visible contact of the peri-implant bone with the implant (Fi) or abutment (Fa) were identified. The distance in millimeters from S to C was defined as the marginal bone level at the crest (MBLc), while the distance from S to Fi was defined as the marginal bone level at the implant (MBLi). The distance between S and Fa was registered as the marginal bone level at the abutment (MBLa). The MBLc distance was considered positive if the C mark was coronal to S, negative if C was apical to it, and as zero if C coincided with the implant shoulder. In the same manner, MBLi was considered negative if Fi was apical to S and as zero if Fi coincided with S or bone–abutment contact was detected. This radiographic analysis was completed by an independent researcher who did not know to which group each implant belonged.

The secondary outcome measures were (a) the implant survival at 4, 8, and 12 weeks and 6 and 12 months, by just clinical examination of the presence, stability, and function of the implant/prosthesis; (b) the implant stability, by means of a comparison of the ISQ values at the corresponding follow-up visits; (c) the PROMs. Finally, three clinical variables were recorded: the modified plaque index (mPLI), the modified sulcus bleeding index (mSBI), and PD, at the mesial, distal, vestibular, and palatal/lingual aspects of the implants at 4 weeks and 3, 6, and 12 months.

### 2.9. Provisional and Definitive Restoration Follow-Ups

The post-loading status of the provisional and definitive restorations was assessed at each follow-up visit. The following complications were registered in the case of occurrence: mobility of the prostheses, mobility of the abutments, abutment screw loosening, the presence of porcelain chippings, the fracture of the frameworks, and the fracture of the abutments.

### 2.10. Adverse Events

Adverse events and complications related to the implant surgical treatment (“implant-related adverse events”) such as acute or chronic pain, sensorial alterations, bone fractures, osteomyelitis, loss of osseointegration of the implant, and discomfort and local or systemic infections were recorded. “Non-implant-related adverse events” such as serious illnesses or any condition requiring hospitalization for more than one day were also recorded.

### 2.11. PROMs (Patient-Reported Outcome Measures)

The PROMs were assessed by means of a questionnaire regarding comfort, esthetics of the restorations, chewing ability, and overall satisfaction. A four-item rating scale was used to assess the degree of satisfaction: excellent, good, fair, and poor.

### 2.12. Statistical Analysis

Sample size was previously calculated with N Query Advisor 4.0 software (Statistical Solutions^®^, Cork, Ireland) using data from previous studies with a similar design [42]. A significance level of 5% (α = 0.05) and a statistical power of 80% were used for the analysis. Radiographic bone changes over time were considered as the primary outcome variable. Considering a mean difference of 0.25 mm of radiographic bone changes as acceptable, a standard deviation of 0.21 mm, and a dropout rate of 10%, a minimum of 20 patients for the overall sample had to be included in the study.

The interclass correlation coefficients to study the consistency among the different consecutive radiographic measurements provided by the same operator on the same patients were significant with a confidence level of 95%, and they all were greater than 0.9, considering 1 as a perfect consistency. Descriptive statistics, such as the means, SEs, SDs, medians, and ranges of the measurements, were calculated for the ISQ, the marginal bone level, and the rest of the clinical values.

Chi-square tests for comparing the categorical variables between two groups were applied. As the variables appeared to be parametric up to the three-month visit, Student’s *t*-tests and ANOVAs were used to analyze parameter evolution and to compare the values between groups. Nonparametric tests such as the Wilcoxon and Mann–Whitney tests were used to compare the data after three months. The mean differences were considered statistically significant at *p* ≤ 0.05 with a confidence interval of 95%. Data analysis was completed with the software package IBM SPSS 21.0^®^ for Windows (IBM, SPSS, Chicago, IL, USA).

Since asymmetry or bias of the data was detected, instead of using the mean and standard deviation, the variables with medians and 25th and 75th percentiles were summarized and the non-parametric Mann–Whitney test was used. The follow-up of the repeated measures was carried out with the non-parametric Friedman test in each of the study groups.

## 3. Results

### 3.1. Study Population

**Figure 2:**
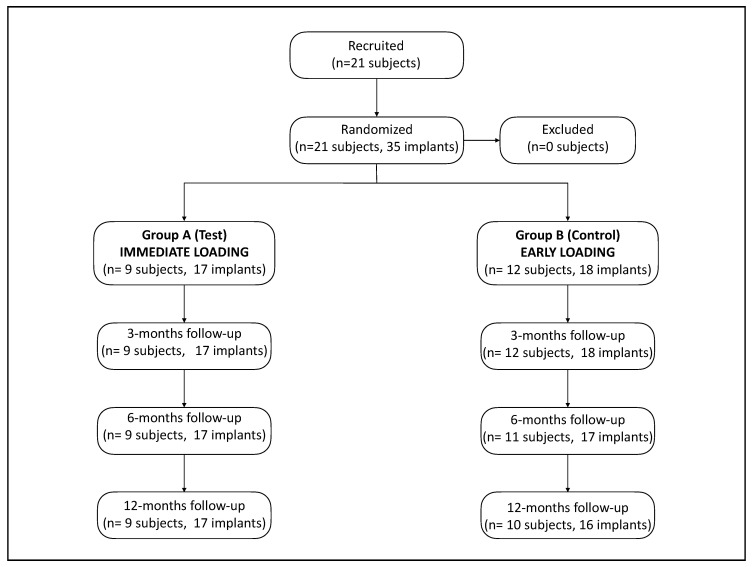
Study sample flow-chart.

Twenty-one patients were recruited in this randomized clinical trial from October 2015 to July 2016. A total of 35 implants were included in the study. No implants were lost, and no implant or non-implant related adverse events were observed throughout the study. Two patients (two implants) were not subjected to X-ray exposure at the six-month visit as they were pregnant at that time. Another two patients (two implants) did not attend the one-year visit. Figure 2 depicts the study sample flow-chart.

### 3.2. Demographic Data

Twelve of the 21 patients (54.2%) were men and nine (45.8%) were women, with a mean age of 52.4 years (SD: 14.6 years). No statistically significant differences between groups were observed for these variables at baseline (*p* = 0.205 and *p* = 0.382 for Groups A and B, respectively). Regarding the smoking habit, five patients (20.8%) were smokers and 19 were not (79.2%). From a periodontal point of view, four patients were healthy (16.9%), and 17 were previously treated: five for gingivitis (20.8%), eight for mild chronic periodontitis (33.3%), six for moderate chronic periodontitis (25%), and one for severe chronic periodontitis (4.2%). Again, no statistically significant differences between Groups A and B were observed for any of the parameters at baseline (*p* = 1 and *p* = 0.470, respectively).

### 3.3. Interventions

**Figure 3:**
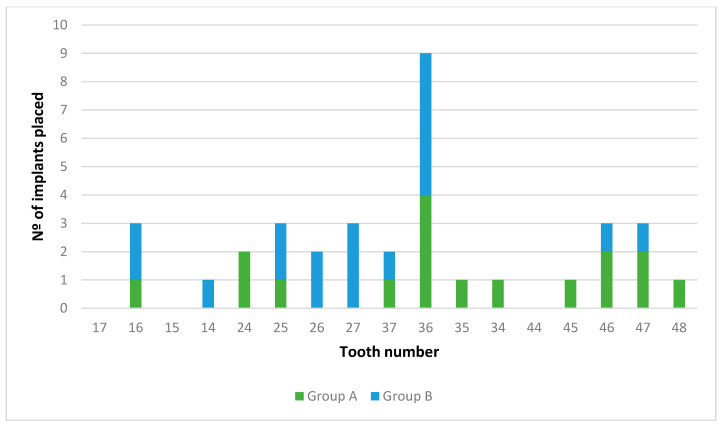
Study sample flow-chart.

Nine patients (42.8%) and 17 implants (48.6%) were assigned to Group A and 12 patients (57.2%) and 18 implants (51.4%) to Group B (early loading). Fourteen implants (40%) were placed in the maxilla and 21 implants (60%) in the mandible. The locations of the implants by tooth site (FDI nomenclature) are displayed in Figure 3. The location more frequent was the left lower first molar. The diameters of the implants used were 3.5 mm (five implants), 4 mm (24 implants), and 4.5 mm (six implants), while the lengths were 8 mm (nine implants), 10 mm (22 implants), and 12 mm (four implants).

Twenty-four implants (68.6%) were restored with single crowns, and 11 implants (31.4%) were part of partial fixed prostheses. Additionally, 52.9% and 47.1% of the implants were restored with single crowns and partial fixed prostheses in Group A, respectively, whereas in Group B, the distribution was 83.3% and 16.7%, respectively. Seven of the abutments placed were of 1 mm in height (five in Group A and two in Group B) and 28 were of 2 mm in height (12 in Group A and 16 in Group B).

**Figure 4:**
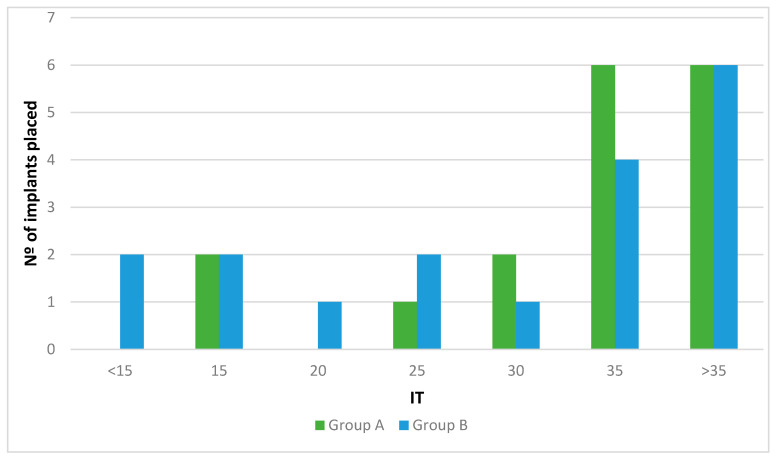
Insertion torque at implant placement.

Regarding the bone quality, 20 implants (57.1%) were inserted into D2 bone (12 and 8 implants in Groups A and B, respectively) and 15 (49.2%) in D3 bone (5 and 10 implants in Groups A and B, respectively). The final IT at the implant placement is shown in Figure 4. No statistically significant differences between groups were observed in any of these variables (*p* > 0.05).

Regarding adverse events, two patients (two implants) of Group B reported minor surgical postoperative acute pain and inflammation that resolved evenly with prescribed medication.

Two patients (two implants) of Group A experienced abutment loosening with provisional restoration at four weeks, and three patients (three implants) of Group B exhibited loosening of the final prosthesis at the three-month visit. The overall survival rate observed at 3, 6, and 12 months was 100% in both groups.

### 3.4. Clinical Variables

Table 1 shows the overall data collection of the clinical variables. There were no significant changes in the mPLI values throughout the study (*p* > 0.05) without differences between groups (*p* > 0.05), nor in the mSBI. An increase in PD (*p* = 0.142), with no differences between groups (*p* = 0.716), was observed throughout the study. However, these changes were not relevant from a clinical point of view.

### 3.5. ISQ measurements

Table 2 presents the overall data collection of the ISQ values. The mean ISQ at the day of the surgery was 74.2 ± 8.34, while it was 69.57 ± 7.90 at four weeks, 70.03 ± 8.94 at eight weeks, 72.99 ± 9.11 at 12 weeks, 75.57 ± 9.88 at six months, and 76.15 ± 10.04 at 12 months. The ISQ evolution by groups is shown in Table 2. Considering the ISQ values of all of the implants at each moment, there were statistically significant differences between groups at 4, 8, and 12 weeks (*p* < 0.05). Analyzing the ISQ values according to the moment of measurement, there were statistically significant differences at four weeks, as well as at 6 and 12 months.

### 3.6. Radiographic Variables: Changes of Marginal Bone Level (CMBL)

Table 3 and Table 4 depicts the CMBL at the crest, the implant, and the abutment over time.

1. Marginal bone level at the crest (MBLc): There were no statistically significant differences between groups (*p* = 0.12). Analyzing the MBLc according to the moment when it was measured, there were statistically significant differences between all surgeries and the rest of the moments of registration (4, 8, and 12 weeks and 6 and 12 months). There were no statistically significant differences (*p* = 0.00) between the MBLc at all follow-up visits.
2. Marginal bone level at the implant (MBLi): There were no statistically significant differences between groups (*p* = 0.195). Analyzing the MBLi according to the moment when it was measured, there were statistically significant differences only between the moment of surgery and 12 weeks. There were no statistically significant differences (*p* = 0.00) between the MBLi at any other moment.
3. Marginal bone level at the abutment (MBLa): There were statistically significant differences between groups (*p* = 0.042). Analyzing the MBLa according to the moment when it was measured, there were statistically significant differences (*p* = 0.024) between the moment of surgery and all of the follow-up visits. Bone contact at the abutment was observed in 51.35% and 61.7% of the implants at 4 and 12 weeks, respectively. These values remained constant after 6 and 12 months with a rate of 63.6% and 67.6%. At the end of the observation period (one year), 66.6% of the implants in Group A and 68.75% in Group B showed bone contact at the abutment. None of these differences were statistically significant.

### 3.7. Patient-Reported Outcome Measurements (PROMs)

**Figure 5:**
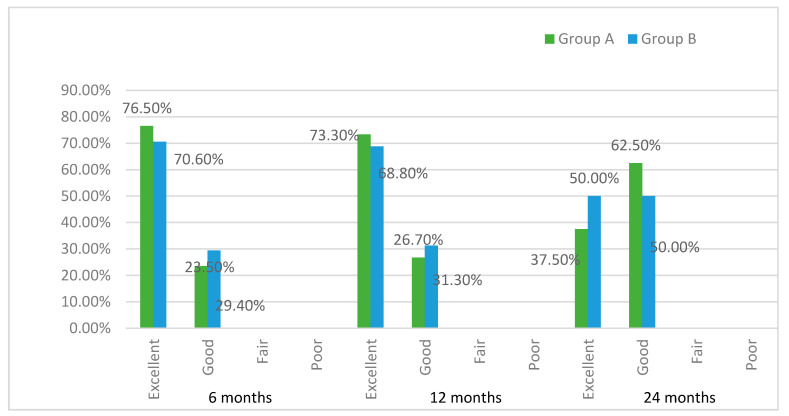
Patient-reported outcome measurements (PROMs).

The overall satisfaction of the patients was high throughout the study and no statistically significant differences were found at 3, 6, and 12 months between groups, as shown in Figure 5.

## 4. Discussion

The outcomes of this randomized clinical trial showed no differences in the survival rate or marginal bone loss, as shown in Table 3, between immediate and early loading protocols of implants located in posterior areas. No implant was lost during the study and the mean bone loss was 0.19 ± 0.37 mm at the implant level and 0.26 ± 0.39 mm at the crestal level (data not shown). These results are in accordance with other clinical trials with a similar design in which high survival rates during the observation period were reported [8,42,43]. The first group did not find any differences in a randomized controlled clinical trial including 72 immediately and early-loaded smooth-collar implants, with a chemically modified surface placed in the posterior mandible [42]. At five years, the survival rate was 100% and the mean bone loss was 0.4 ± 0.24 mm and 0.8 ± 0.15 mm in test and control groups, respectively. Similarly, a recent investigation on tissue-level implants with an SLActive^®^ surface showed survival rates of 97.4% and 96.7% and mean bone level changes of 0.88 ± 0.81 mm and 0.57 ± 0.83 mm for immediately and early-loaded groups, with no significant differences between them [8]. The thermo-chemically modified implant surface used in the present study has shown highly osseoconductive properties in in vivo studies with a mean bone to implant contact of 80% at three weeks [28,29,44]. This may have contributed to the high survival rates of the implants under immediate and early loading conditions in posterior areas. The predictability of early-loaded, platform-switched implants with a similar hydrothermally modified surface has been recently demonstrated in a three-year randomized clinical trial in which two types of surfaces were compared [11]. The function of the implants was restored after 10–14 weeks and a 100% survival rate after three years was attained in both groups with no differences in marginal bone loss (1.12 ± 0.49 mm vs. 1.10 ± 0.38 mm). Immediate and early loading after three weeks with hydrothermally treated hydroxyapatite implants with a platform-matching connection were investigated in a prospective clinical study [30]. The survival rate was 100% and 94.7% for the immediately and early-loaded groups, respectively, and the mean bone loss was 0.75 ± 0.50 mm with no differences between groups after two years of function. In a randomized clinical trial with platform-switched implants, no differences in the survival rates between immediate and early loading at three weeks and after three years were observed [38]. The immediately loaded implants lost 0.42 ± 0.59 mm and 0.90 ± 1.17 mm of peri-implant bone at one and three years, respectively; for the early-loaded implants, bone loss was 0.46 mm (95% CI: 0.20, 0.72) and 1.10 ± 1.39 mm at the same time points. In the present study, the final marginal bone loss at the implant level was 0.12 ± 0.23 mm and 0.27 ± 0.26 mm for the immediately and early-loaded groups, respectively, at one year.

The current literature has reported some differences in the maintenance of peri-implant bone with different implant designs [45]. In fact, platform-switched implants and conical internal connections obtain better results compared to platform-matching and external connection implants [46,47,48]. The biological response to platform-switching implants with a conical connection has recently been investigated in a dog model [49]. It was observed that at platform-switched implants, the connective tissue was located coronally with respect to the implant–abutment connection, and the epithelium was never found below this level, unlike at matching-platform implants. From a biological point of view, this means that the epithelial attachment can be kept far from the microgap and only a minimum bone resorption may be expected after healing.

In order to minimize bone resorption, implants with a conical internal connection and a platform-switching design were used in the present investigation. In addition, definitive abutments were tightened immediately after implant placement according to the “one abutment–one time” protocol [37,39,50]. In this sense, Molina et al. compared platform-switched implants with one-time abutment placement or implants in which repeated connections/disconnections were performed, and found statistically significantly less bone resorption at six months post-loading between the test (0.61 ± 40 mm) and control (1.24 ± 0.79 mm) groups [39].

In the present trial, the implants were inserted at least 1 mm subcrestally, as evidenced by the radiographic distance from the top of the bone crest to the implant shoulder (mean MBLc: 1.06 ± 0.52 mm, data not shown). Whether the subcrestal position of an implant can influence the rate of marginal bone loss is still a matter of discussion [51,52,53]. Recent reports suggest that it may lead to better maintenance of peri-implant bone [54,55]. Fetner et al. investigated the differences between the subcrestal and equicrestal positions of the implant shoulder in an in vivo study [53]. Similar rates of bone loss at the crest were shown between groups, but there was increased bone loss at the implant in the equicrestal group. The histology showed bone contact at the abutment in the subcrestal group, while, in any case, this phenomenon was observed in the equicrestal group. These results have been confirmed by other clinical and histological studies [56,57]. A histomorphometric evaluation of retrieved human implants showed that all subcrestal implants presented bone contact at the abutment, while 0.5–1.5 mm bone loss was observed at equicrestal implants [56].

Within the limitations of the radiographic evaluation method used in the present study, bone contact at the abutment was observed in 68.75% of the implants at one year. In Group A, an increase in the bone–abutment contact during the study was shown (from 52.9% at one month to 68.75% at one year). The same trend was observed in the test group, from 58.8% at one month to 66.6% at one year) (data not shown). Whether these findings are related to the different positions of the implant shoulders (MBLc) at baseline between the two groups (0.87 ± 0.59 mm in Group A and 1.25 ± 0.39 mm in Group B) or with the different loading protocols (immediate versus early), or both, is something that cannot be answered under this protocol. In this way, Koutozis et al. and Degidi et al. demonstrated a direct correlation between the depth of the implant shoulder and the bone–abutment contact with platform-switched implants [56,57]. However, no association was found between the subcrestal position of the implant and peri-implant bone loss over time (CMBLi), which is in accordance with previous findings [52,55]. Differences in bone loss at the implant were demonstrated comparing the equi- and subcrestal positions of Ankylos^®^ implants in another clinical trial; however, a higher bone resorption at the crest for subcrestal implants was showed in a radiological CBCT evaluation [57]. Bone loss at the crest was 0.08 ± 0.25 mm, 0.65 ± 0.45 mm, and 0.85 ± 0.75 mm at the equicrestal position, 1 mm subcrestal position, and 2 mm subcrestal position, respectively, at one year. Degidi, in 2017, with the same type of implants, reported a bone loss at the crest of 0.42 ± 0.77 mm after three years [58].

In our research, the implant level at the time of surgery was at least 1 mm below the bone margin. As seen in the Table 3, at 4 weeks and 3, 6, and 12 months, a moderate mean bone loss could be observed, in concordance with the abovementioned studies. At one year, the mean bone loss considering all implants was 0.26 ± 0.39 mm, and 0.21 ± 0.28 mm and 0.31 ± 0.49 mm for immediate and early loading, respectively. Significant differences could not be found at any moment of the study. These results suggest that the rate of bone loss at the crest seems to be similar between the subcrestal and equicrestal positions of the implant. However, a deeper position of the implant margin could be recommended in order to avoid coronal exposure of the implant body, which could favor the apparition of mucositis [59].

The available literature suggests that primary stability is a relevant factor that may influence the osseointegration of immediately loaded implants [4,60,61,62,63]. The ISQ value at the abutment level, insertion torque, and manual stability were used to assess the primary stability in this study. Resonance frequency analysis is considered the most reliable method to evaluate implant stability over time after implant placement [64,65,66,67,68].

In order to avoid unscrewing the abutments, and with respect to the “one-abutment–one time” concept, the ISQ value was registered at the abutment level, discarding the ISQ at the implant level. A common finding when assessing implant stability in clinical trials is the initial drop in ISQ values within the first two to four weeks of healing after implant placement [69,70,71], which is caused by osteoclastic bone resorption [72]. Although it could be argued that chemical modification of the surface could somehow reduce this phenomenon, due to accelerated bone formation around the implant, a slight decrease in ISQ after surgery was still found in some studies using similar surfaces, especially with high ISQ values [73,74]. This is consistent with the findings of our study, in which the overall mean value dropped moderately from surgery to four weeks (74.2 ± 8.4 to 70 ± 8.9; data not shown), although not statistically significant. Unfortunately, in this study, the ISQ values were not recorded at two weeks, so we were not able to determine whether there was a reduction in implant stability at this time. After four weeks, the ISQ values kept increasing up to the one-year visit in a similar manner in both groups. A progressive increase in implant stability after complete osseointegration was also observed by Kokovic et al., who compared immediate and early loading in 72 self-tapping implants with an SLA^®^ surface, finding a progressive increase in the ISQ values from surgery up to five years [42]. In a similar way, the immediately loaded group at our study showed significantly higher stability over time compared to the early-loaded group. The overall values can be examined in Table 2.

Across the study period, implant stability in the immediately loaded group was higher compared to the early-loaded group. However, this difference could be associated with a higher proportion of implants placed in the mandible and the D2 bone quality of Group A. Indeed, a positive association between higher ISQ values and mandibular bone (*p* = 0.002) and D2-type bone (*p* = 0.003) was observed in the study. These results are in agreement with an investigation where higher ISQ values of implants were found at the time of surgery and at two, four, and eight weeks in areas with bone type D2 compared to bone types D3 and D4 [75]. This correlation between the presence of cortical bone and a higher primary stability has been well documented already in the literature [72,76,77,78].

Another interesting finding of the present research was the negative association between ISQ values at the time of surgery and the insertion torque. Lower values of insertion torque were not associated with lower ISQ values. In this way, the correlation between insertion torque and resonance frequency analysis measurement is still controversial in the literature [73,79,80,81]. One possible explanation could be the design at the neck of the implants used in this study. These implants have an inverted conical shape at their coronal part, leaving a horizontal space between the implant shoulder and the bone that could be filled by the coagulum [82]. Once the implant shoulder has been inserted in an equicrestal position, implants tend to lose rotatory stability, especially in thin cortical bone. However, a subcrestal position of the implant tends to increase the ISQ values [83,84,85,86,87] and this could explain the high ISQ values found at the time of surgery. Although this was not an objective of the present study, it could also be highlighted from this investigation that implants with splinted prostheses showed a significant increase in their ISQ values when compared to single crowns at one year (*p* = 0.042). This finding has not been reported in other similar trials [8,42,88]. The relationship between crowns splinting on implants and implant stability still remains unclear.

A limitation of this study was the use of standardized intraoral radiographs to assess marginal bone level, even though this is a well-validated method in the literature [89], since buccal and lingual bone could not be evaluated. Furthermore, the first bone to abutment contact point of the implants was considerably challenging to visualize and interpret with the used method. On the contrary, this reduced the radiation exposure of patients compared to cone beam computed tomography.

Additionally, the preliminary results of this randomized clinical trial should be interpreted with caution due to the limited sample size and follow-up.

## 5. Conclusions

Within the limitations of this study, and in relation to the primary objective of this research, it could be concluded that immediate (one week) or early (four weeks) loading of implants a with thermo-chemically treated surface, under the concept of “one abutment–one time,” does not influence the marginal bone level at one year. Regarding the secondary objectives, neither treatment modalities influenced the peri-implant condition, the implant stability over time, or the PROMs. This study suggests that both loading protocols with the mentioned implants and conditions could be considered as treatment options for restoring the posterior areas of the maxilla and mandible. However, these results have to be confirmed by further studies with a larger sample and longer follow-up periods.

## Figures and Tables

**Table 1:** Clinical variables. mPLI, modified plaque index; mSBI, modified sulcus bleeding index; PD, probing depth.

		A, Test (IMMEDIATE LOADING)		B, Control (EARLY LOADING)		*p*-Values
		Mean	SD	Mean	SD	
mPLI	4 weeks	0.13	0.25	0.08	0.17	0.590
	3 months	0.37	0.59	0.08	0.22	0.807
	6 months	0.09	0.22	0.06	0.11	0.892
	12 months	0.07	0.12	0.16	0.29	0.736
mSBI	4 weeks	1.44	0.60	1.25	0.51	0.424
	3 months	1.31	0.35	1.17	0.28	0.219
	6 months	1.13	0.25	1.30	0.38	0.259
	12 months	1.16	0.31	1.22	0.50	0.345
PD	4 weeks	1.96	0.53	2.00	0.78	0.845
	3 months	2.13	0.65	1.79	0.65	0.132
	6 months	2.04	0.57	2.15	0.69	0.990
	12 months	2.00	0.74	2.25	0.50	0.078

**Table 2:** Implant quotient stability (ISQ) values at the time of surgery, at 4 and 8 weeks, and at 3, 6, and 12 months. * Statistically significant differences between Groups A and B (*p* < 0.05). ^a–k^ Statistically significant differences between values with the same letter (*p* < 0.05).

	A, Test (IMMEDIATE LOADING)					B, Control (EARLY LOADING)					*p*-Values
	Mean	Median	Asymmetry	25th Percentile	75th Percentile	Mean	Median	Asymmetry	25th Percentile	75th Percentile	
Surgery	72.03 ^d,e^	70.00	0.53	65.50	80.00	67.25 ^h,i^	66.00	1.31	62.75	68.38	0.072
4 weeks	73.29 ^a,b,c^	70.50	0.68	64.50	79.50	66.94 ^f,g^	62.25	1.11	63.38	68.25	0.041 *
8 weeks	76.56	74.00	0.72	68.75	83.75	69.61 ^j^	68.75	1.97	65.88	69.63	0.014 *
3 months	77.18 ^a^	72.00	0.49	69.25	84.00	70.67 ^k^	70.00	1.68	67.00	71.50	0.049 *
6 months	78.88 ^c,e^	73.50	0.50	70.00	89.5	72.27 ^f,h,j^	70.00	1.79	68.00	73.00	0.062
12 months	79.35 ^b,d^	73.00	0.38	70.00	88.25	72.75 ^g,I,k^	70.25	1.70	67.25	74.00	0.870

**Table 3:** Radiographic variables. * *p* ≤ 0.05. Marginal bone level at the crest (MBLc). Marginal bone level at the abutment (MBLa).

		A, Test (IMMEDIATE LOADING)		B, Control (EARLY LOADING)		*p*-Values
		Mean	SD	Mean	SD	
MBLc	Surgery	0.87	0.59	1.25	0.39	0.03 *
	4 weeks	0.60	0.58	0.89	0.54	0.14
	12 months	0.51	0.63	0.76	0.70	0.28
MBLa	Surgery	0.00	0.00	0.00	0.00	1

**Table 4:** Mean, Median & Percentile of the radiographic variables. * *p* > 0.05. Marginal bone level at the crest (MBLc). Marginal bone level at the implant (MBLi). Marginal bone level at the abutment (MBLa).

		A, Test (IMMEDIATE LOADING)					B, Control (EARLY LOADING)					*p*-Values
		Mean	Median	Asymmetry	25th Percentile	75th Percentile	Mean	Median	Asymmetry	25th Percentile	75th Percentile	
MBLc	3 months	0.53	0.67	0.07	0.08	0.85	0.92	0.75	1.39	0.58	1.20	0.089
	6 months	0.67	0.70	0.04	0.23	1.15	0.81	0.62	1.44	0.42	1.06	0.882
MBLi	Surgery	–0.03	0.00	–3.14	0.00	0.00	0.00	0.00	0.00	0.00	0.00	0.386
	4 weeks	–0.10	0.00	–2.46	–0.02	0.00	–0.02	0.00	–2.71	0.00	0.00	0.557
	3 months	–0.14	–0.08	–1.69	–0.22	0.00	–0.03	0.00	–2.90	0.00	0.00	0.074
	6 months	–0.12	0.00	–1.89	–0.18	0.00	–0.07	0.00	–3.31	–0.07	0.00	0.309
	12 months	–0.12	0.00	–2.48	–0.10	0.00	–0.27	0.00	–1.75	–0.57	0.00	0.794
MBLa	4 weeks	0.45	0.27	0.96	0.00	0.81	0.37	0.22	1.09	0.00	0.69	0.658
	3 months	0.13	0.00	0.78	0.00	0.25	0.31	0.22	2.50	0.00	0.45	0.182
	6 months	0.24	0.20	0.88	0.00	0.42	0.31	0.00	2.46	0.00	0.46	0.657
	12 months	0.21	0.11	1.89	0.00	0.39	0.31	0.16	2.57	0.00	0.37	0.657

## Data Availability

The data presented in this study are available on request from the corresponding author. The data are not publicly available due to privacy.
